# Editorial: The role of faith in the mental health and integration of forcibly displaced populations

**DOI:** 10.3389/fpsyt.2025.1640507

**Published:** 2025-07-08

**Authors:** Diana Rayes, Courtland Robinson, Ayesha Ahmad, Alastair Ager

**Affiliations:** ^1^ Department of International Health, Johns Hopkins University, Baltimore, MD, United States; ^2^ Global Health Humanities, St George’s University of London, London, United Kingdom; ^3^ Institute for Global Health and Development, Queen Margaret University, Edinburgh, IN, United States; ^4^ Mailman School of Public Health, Columbia University, New York, NY, United States

**Keywords:** mental health, refugees & asylum seekers, integration, faith, forced displacement & migration

In contexts of armed conflict and forced displacement, the erosion of safety, identity, and belonging often intersects with significant challenges to mental health and psychosocial well-being. Nearly 120 million individuals – equivalent to 1.5% of the global population – are estimated to have experienced forced displacement (1). For many of these individuals, faith is not a peripheral concern, but a central, organizing force in their lives. Yet, mental health research and interventions in humanitarian settings have largely failed to adequately incorporate or acknowledge the profound role of religion and spirituality in shaping how displaced individuals interpret suffering, seek support, and imagine healing (2, 3).

This timely Research Topic brings together studies that illuminate the complex and deeply rooted role of faith in the mental health journeys of displaced populations. The contributions span diverse geographies and methodologies, but collectively point to a single, urgent insight: faith is not only a source of individual and communal resilience but also a dimension of health that humanitarian response systems must acknowledge and better accommodate.

Rutledge’s mixed methods study explores both gender- and faith-based coping mechanisms among internally displaced Sunni Muslim women in northern Iraq. It offers deep insight into how faith structures daily resilience of these individuals. Critically, the study highlights a mismatch between humanitarian services and the spiritual needs of displaced communities which, if neglected, can profoundly deepen suffering. The paper thus concludes with strong recommendations to better integrate gender- and faith-sensitive resources in psychosocial interventions provided in these settings.

Bridi et al. take a life-course, qualitative approach to identify how faith shapes understandings of mental illness and dementia among aging Arab refugees in Jordan and the United States. Participants shared openly how faith and spirituality play in the epistemologies of illness and healing, suggesting the importance of tailoring public health and clinical interventions to meet the spiritual needs of this population. Their findings expand the concept of resilience beyond periods of crisis, suggesting that faith remains a sustaining force across the aging process.

El-Khani et al. explore the social ecological level of the family, using the United Nations’ Office on Drugs and Crime (UNODC)’s implementation of the *Strong Families* program across more than 30 countries to reflect on the role of faith within a family structure in building and fostering resilience. The authors emphasize four key principles: recognizing local religious practices, ensuring materials are faith-appropriate, avoiding forced discussions of belief, and training facilitators to respectfully engage with questions of faith. Challenging traditionally secular humanitarian frameworks, the authors argue for an approach that integrates religious literacy into program design in order to foster trust between aid providers and beneficiaries and improve mental health outcomes.

However, sociopolitical conditions can often constrain the protective potential of faith in crisis contexts. Albahsahli et al. explore this in their study of the downstream impacts of the “Muslim” ban in the United States on Iraqi and Syrian refugees with hypertension, illustrating the relevance of social ecological analysis at the societal level. Beyond the immediate stressors of their forced displacement and chronic illness, participants emphasized how discrimination, Islamophobia and family separation introduced new layers of trauma and even exacerbated their chronic diseases. This speaks to the impacts that exclusionary policies may have on the very methods that individuals may rely on, including those entrenched in frameworks of faith or spirituality, to cope with adversity.

Taken together, these thought-provoking studies suggest that faith functions at multiple levels: as a long-term resource of *individual* meaning-making and navigating cognitive and emotional aging and chronic illness; as a *family- and community-based* framework for parenting and caregiving; and as a lifeline in *social systems* that are exclusionary or discriminating.

Yet humanitarian systems have often marginalized expressions of faith or spirituality, treating it as irrelevant or irrational (4). The consequences, as demonstrated by the perspectives shared by participants across the studies featured in the Research Topic, are significant. When faith is ignored or dismissed, interventions targeting mental health or psychosocial outcomes may fail to resonate with affected communities, and may inadvertently deepen feelings of distrust or alienation. On the other hand, when faith is integrated thoughtfully and respectfully into programs and service delivery, it can become a powerful tool for healing and connection (5).

These studies thus contribute to the growing evidence that points to the importance of promoting religious literacy throughout mental health and psychosocial support (MHPSS) interventions targeting communities in crisis (3). For example, providers must be trained to understand how faith intersects with mental health, such as how trauma can rupture or reinforce religious belief or how faith-based/spiritual practices can be drawn on to build resilience and promote recovery. Humanitarian systems should also be mindful of both programs and policies that may be exclusionary or discriminatory to members of various faith groups by nature.

This small, yet significant body of work also challenges the universality of Western approaches to treating mental health and psychosocial needs. For many displaced populations, the self is inseparable not only from collective systems, such as family or community, but also from a sense of the sacred and divine shared (and, on occasion, contested) within these collectives. Promoting good mental health is thus not just about reducing symptoms, but also about building resilience and the communal capacity to endure suffering with dignity and meaning, allowing for better integration in their new, perhaps temporary, contexts.

In summary, this Research Topic affirms the centrality of faith in the lived experience of displacement and mental health. It calls for a paradigm shift: one in which faith is not sidelined but considered non-exceptional, and thus actively engaged with in the design of interventions that promote positive mental health and prevent mental illness, the training of providers, and the shaping of humanitarian policies and systems. As the global displacement crisis continues to grow, and with it the burden of poor mental health, faith-informed approaches offer cultural relevance and profound potential for healing and hope.
